# Gross anatomy performance associated with human cadaver dissection compared to non-cadaveric methods in nigerian medical students: a comparative cross-sectional study

**DOI:** 10.1186/s12909-026-09566-0

**Published:** 2026-05-29

**Authors:** Willy Barinem Vidona, Collins Nduka Esomchi, Mariam Adurelere Rabiu

**Affiliations:** 1 Department of Anatomy, Faculty of Basic Medical Sciences, Ambrose Alli University, Ekpoma, Edo State Nigeria; 2 Department of Human Anatomy, Faculty of Basic Medical Sciences, State University of Medical and Applied Sciences, Igbo Eno, Enugu State Nigeria

**Keywords:** Cadaver dissection, Gross anatomy, Medical education, Anatomy teaching methods, Nigerian medical students, Knowledge outcomes

## Abstract

**Introduction:**

Gross anatomy forms the foundation of medical education, yet traditional cadaver dissection faces challenges from cadaver shortages, costs, and curriculum changes, prompting increased use of alternatives such as anatomical models, virtual dissection software, and textbooks. Evidence on their comparative effectiveness remains mixed, particularly in resource-limited settings. This study compared gross anatomy examination performance between medical students exposed to human cadaver dissection and those taught using only non-cadaveric methods in a Nigerian setting.

**Methods:**

This comparative cross-sectional study involved 120 first-year medical students at Ambrose Alli University, Nigeria. Participants were grouped according to their actual practical teaching exposure during extremity anatomy training: Group A (*n* = 69, cadaver dissection) and Group B (*n* = 51, non-cadaveric methods only). Performance was assessed using identical institutional final examinations consisting of 50 MCQs and essay questions. Data were analysed with descriptive statistics, Pearson correlation, independent t-tests (Welch’s correction), Cohen’s d, and chi-square tests.

**Results:**

Students in Group A achieved higher mean scores than Group B in MCQs (69.90% vs. 55.79%), essays (61.16% vs. 52.92%), and total scores (65.00% vs. 54.82%; all *p* < 0.001), with large effect sizes (Cohen’s d = 0.70–1.09). The cadaver dissection group also had higher distinction rates and lower failure rates.

**Conclusion:**

In this cohort, exposure to human cadaver dissection was associated with higher gross anatomy examination performance compared to non-cadaveric methods alone. While these findings suggest that cadaver-based teaching remains valuable in resource-limited settings, the observational design limits causal inference. Further multi-institutional studies are warranted.

**Supplementary Information:**

The online version contains supplementary material available at 10.1186/s12909-026-09566-0.

## Introduction

Gross anatomy remains the cornerstone of medical education [1], providing medical students with essential three-dimensional knowledge of human structure, spatial relationships, tissue textures, and clinical correlations critical for diagnosis, imaging interpretation, surgical procedures, and safe patient care [2]. Deficiencies in anatomical knowledge have been associated with increased risk of medical errors, particularly in procedural specialties [3].

Human cadaver dissection has long served as the traditional gold standard for teaching gross anatomy [4]. It offers unparalleled hands-on experience, enabling students to explore layered structures, appreciate tissue properties through tactile feedback, and observe natural anatomical variations that textbooks and idealized models cannot fully replicate [3, 5]. Anatomical variations are common, with many structures showing variation in a substantial proportion of individuals [6]. Cadaveric dissection exposes learners to biological diversity in a real-world setting, enhancing critical thinking and connecting textbook anatomy with clinical reality [5]. In addition, cadaver dissection is believed to contribute to the development of professionalism, empathy, and ethical awareness [7].

However, cadaver-based teaching faces substantial challenges globally, including cadaver shortages, high procurement and preservation costs, ethical and religious concerns, and reduced curricular time [5, 8]. Many institutions have therefore adopted alternative teaching methods such as anatomical models, prosections, plastinated specimens, virtual dissection software, three-dimensional printed models, and augmented reality platforms [9, 10].

Recent studies show mixed findings regarding their comparative effectiveness. Some report advantages of virtual or model-based methods in knowledge gains, student satisfaction, and accessibility [3, 9–11], while others suggest traditional dissection offers superior benefits in spatial reasoning, three-dimensional understanding, and clinical application [12]. Overall, systematic reviews highlight that no single method consistently demonstrates clear superiority across all outcomes, with results often varying by assessment type and anatomical region [13, 14].

These challenges are particularly acute in low- and middle-income countries, especially in sub-Saharan Africa and Nigeria. Cadaver scarcity remains a major barrier, often relying on unclaimed bodies that result in inconsistent supply, poor preservation quality, and gender imbalances [15]. Voluntary body donation is rare due to religious beliefs, cultural taboos, and a lack of supportive legislation [16]. Additional constraints, such as inadequate storage facilities, absence of plastination technology, large class sizes, limited practical time, and insufficient funding, compromise hands-on learning and contribute to overloaded curricula [5, 8]. Despite these difficulties, many African students and faculty continue to value cadaver dissection when available, viewing it as superior for regional anatomy comprehension and professional development [12, 17, 18].

While the global literature provides useful insights, empirical comparative studies using objective summative examination outcomes from resource-constrained African settings remain limited. Most evidence originates from high-income countries or focuses on student perceptions rather than measurable academic performance. The present study, therefore, aimed to compare gross anatomy examination performance between first-year medical students with exposure to human cadaver dissection and those taught solely using non-cadaveric methods in a Nigerian setting. It was hypothesised that students with cadaver dissection experience would show higher performance in standardised assessments compared to those using non-cadaveric methods alone.

## Materials and methods

### Study design

This was a comparative cross-sectional study that evaluated the association between exposure to human cadaver dissection and gross anatomy knowledge outcomes among medical students. The study compared examination performance between students who had received cadaver-based teaching and those who had received only non-cadaveric teaching methods during their first-year gross anatomy course.

### Participants and sampling

The study was conducted at Ambrose Alli University, Ekpoma, Edo State, Nigeria. Participants were medical students who had completed their first-year gross anatomy curriculum (extremity anatomy) during the 2023/2024 academic session. A convenience sampling technique was employed due to logistical constraints and limited resources. Questionnaires seeking consent to access institutional examination records were distributed to 250 students who were readily accessible during lecture and practical sessions. A total of 120 students returned fully completed consent forms and were included in the analysis (response rate = 48%).

### Teaching exposure and group allocation

Due to an acute shortage of human cadavers during the semester dedicated to extremity anatomy (upper and lower limbs), the Department of Anatomy divided students into practical batches based on matriculation numbers and existing lecture group schedules. This administrative allocation resulted in two naturally occurring groups:

- Group A (Cadaver Dissection Group, *n* = 69): Students received approximately 60 h of supervised human cadaver dissection (2 sessions per week, 2 h per session). Students performed active dissection on formalin-preserved cadavers sourced primarily from unclaimed bodies. The student-to-cadaver ratio was approximately 10–15 students per cadaver. The source and procurement context of the cadavers were not formally disclosed to students, although they were informed at the start of the course about the respectful use of unclaimed bodies for education.
- Group B (Non-Cadaver Group, *n* = 51): Students received an equivalent of 60 h of supervised practical teaching (also 2 sessions per week, 2 h per session). The student-to-resource ratio was as follows:
  i. Anatomical models and plastinated specimens: approximately 6–8 students per station.
  ii. Virtual dissection software (Complete Anatomy and 3D Atlas): 1–2 students per computer workstation.
  iii. Textbooks and atlases: used in small groups of 4–6 students.

Although plastinated specimens are cadaver-derived, students did not perform active wet dissection.

Both groups attended the same lectures delivered by the same instructors and followed identical learning objectives. Students in both groups had access to shared departmental resources (textbooks, atlases, and limited virtual software). Access to resources outside the assigned practical teaching modality was not strictly monitored or restricted. In the subsequent semester, a crossover was implemented so that all students eventually experienced both teaching modalities. This study reports findings from the first semester only.

### Assessment instruments

Both groups sat for the identical institutional final examination in gross anatomy (upper and lower extremities). The examination was blueprint-based and mapped to the course learning objectives. It included 50 MCQs (maximum 100 marks) and essay questions. Approximately 30–40% of MCQs and most essay questions incorporated clinical vignettes to assess higher-order cognitive skills (application and analysis levels according to Bloom’s taxonomy). Some MCQs were negatively phrased. Items were previously used in the department with minor modifications. Faculty members who marked the examinations were blinded to group allocation. Scores were expressed as percentages and categorised according to Medical and Dental Council of Nigeria and National Universities Commission guidelines: fail (< 50%), pass (50–69%), distinction (≥ 70%). While content validity was ensured through alignment with curriculum objectives, formal psychometric data (e.g., difficulty and discrimination indices) from previous administrations were not available for this study, and no post-hoc item analysis was performed. The MCQs and essay questions used in the examination are provided in Appendix 1 for reference.

### Data collection

Data collection occurred in two parts. A structured questionnaire was administered to obtain written informed consent and basic demographic information (age, sex, and previous academic performance in related courses).

The primary outcome data (gross anatomy examination scores) were obtained directly from official institutional records of the first-year final examination, rather than through self-report. This approach ensured accuracy and eliminated recall bias. The questionnaire served only as a tool to link participant consent with their official examination results.

Official examination scores were retrieved from institutional records. The questions used in the examination are presented in Appendix 1.

### Ethical considerations

The study received ethical approval from the Research Ethics Committee of Ambrose Alli University, Ekpoma (Clearance No. 012/25) and was conducted in accordance with the Declaration of Helsinki. Written informed consent was obtained from all participants for the use of their examination scores and anonymised data. The use of human cadavers in teaching at the institution complies with national ethical and legal guidelines. This was an observational study of naturally occurring teaching conditions caused by severe cadaver scarcity. Although the crossover in the subsequent semester helped promote eventual equity, we acknowledge that unequal access to cadaver dissection during the assessed semester created temporary educational inequity within the same cohort. This situation reflects the difficult realities faced by many anatomy departments in resource-limited settings. The study did not involve any researcher-imposed withholding of educational opportunities.

### Statistical analysis

Data were analysed using SPSS version 25. Score distributions were visually inspected using histograms and Q-Q plots and formally tested for normality using the Shapiro-Wilk test. Although some mild deviations from normality were observed, parametric tests were used because they are robust with the current sample size (*n* = 120) and produced results consistent with non-parametric alternatives. Descriptive statistics (means, standard deviations, medians, modes, and ranges) were calculated for examination scores. Baseline characteristics between groups were compared using independent samples t-tests for continuous variables and chi-square tests for categorical variables. Pearson correlation coefficient was used to assess the relationship between MCQ and essay scores. Independent samples t-tests with Welch’s correction for unequal variances were performed to compare mean scores between Group A and Group B. Mean differences, 95% confidence intervals, and Cohen’s d effect sizes were reported. Chi-square tests of independence were used to compare the distribution of performance categories (distinction, pass, and fail) between the two groups. A two-tailed *p*-value of < 0.05 was considered statistically significant.

## Results

A total of 120 medical students participated in the study (Group A: *n* = 69; Group B: *n* = 51). The two groups were comparable in baseline characteristics (Table 1).

**Table 1:** Baseline characteristics of study participants

Characteristic	Group A (Cadaver) (*n* = 69)	Group B (Non-Cadaver) (*n* = 51)	*p*-value
Age (years) (Mean ± SD)	21.7 ± 2.2	21.9 ± 2.1	0.52
Sex, *n* (%)			0.75
Male	41 (59.4)	29 (56.9)	
Female	28 (40.6)	22 (43.1)	
Previous Cumulative Grade Point Average (Mean ± SD)	4.43 ± 0.50	4.38 ± 0.48	0.46

There was a significant positive correlation between MCQ and essay scores across all participants (Pearson’s *r* = 0.62, *p* = 0.01).

Descriptive statistics of the examination scores are presented in Table 2.

**Table 2:** Descriptive statistics of examination scores for group A and group B

Statistic	MCQ Group A	MCQ Group B	ESSAY Group A	ESSAY Group B	TOTAL Group A	TOTAL Group B
Mean	69.90	55.79	61.16	52.92	65.00	54.82
Median	74.00	58.00	62.00	53.00	67.50	54.20
Mode	82.00	48.00	54.00	59.50	59.50	44.50
Std. Dev	12.24	13.90	11.42	12.27	10.66	11.90
Minimum	30.00	26.00	35.00	26.00	28.00	29.55
Maximum	87.00	82.00	85.00	77.00	79.50	76.50

Comparison of mean scores between the two groups is shown in Table 3.

**Table 3:** Independent samples t-test results (welch’s correction)

Pair	Mean Difference	95% CI	t	df	*p*-value	Cohen’s d
MCQ (A vs. B)	14.11	8.40–19.82	4.94	90.3	< 0.001	1.09
ESSAY (A vs. B)	8.24	3.93–12.55	3.81	106.8	< 0.001	0.70
TOTAL (A vs. B)	10.18	6.10–14.26	4.94	97.5	< 0.001	0.91

The frequency distribution of performance categories for MCQ, Essay, and Total scores is presented in Table 4.

**Table 4:** Frequency distribution of performance categories

Category	Group A (*n* = 69) n (%)	Group B (*n* = 51) n (%)
MCQ		
≥ 70 (Distinction)	42 (60.9)	15 (29.4)
50–69 (Pass)	23 (33.3)	33 (64.7)
< 50 (Fail)	4 (5.8)	3 (5.9)
Essay		
≥ 70 (Distinction)	16 (23.2)	4 (7.8)
50–69 (Pass)	45 (65.2)	31 (60.8)
< 50 (Fail)	8 (11.6)	16 (31.4)
Total		
≥ 70 (Distinction)	28 (40.6)	9 (17.6)
50–69 (Pass)	36 (52.2)	27 (52.9)
< 50 (Fail)	5 (7.2)	15 (29.4)

## Discussion

This study provides empirical evidence from a Nigerian medical education context that exposure to human cadaver dissection leads to higher examination scores compared to non-cadaveric methods, such as anatomical models, virtual dissection software, and textbooks. In this comparative cross-sectional study, first-year medical students who participated in cadaver dissection practicals scored higher on gross anatomy examinations than those taught with only non-cadaveric methods during the same semester. The cadaver dissection group showed consistently higher mean scores across MCQ, essay, and total assessments, with large effect sizes and improved performance categories.

These findings are consistent with the broader literature suggesting that active cadaver dissection may offer advantages in developing three-dimensional spatial relationships and integrative anatomical understanding, particularly when assessments include essay-type questions that require relational and clinical reasoning [19, 20]. For example, comparative investigations in high-resource settings have shown dissection groups outperforming model- or virtual-only groups on practical and written assessments of complex structures [21, 22]. However, they contrast with reports showing comparable or superior outcomes with virtual dissection tools and models in specific contexts, especially for immediate knowledge gains and student satisfaction [13, 23]. Differences in findings may be related to assessment formats, anatomical regions studied, and the specific resources available to each group. The present study did not assess student satisfaction, so direct comparisons on this outcome should be interpreted with caution.

The findings strongly support the adoption of hybrid anatomy teaching models in resource-constrained environments [24]. Strategic integration, such as using virtual platforms and models for preparation and reinforcement while reserving limited cadaver time for complex relational anatomy and tissue texture appreciation, may offer the best balance [19, 25]. Such hybrid approaches are increasingly recommended as realistic solutions in different settings, especially in low- and middle-income countries [26, 27].

Within the context of this Nigerian medical school, where cadaver availability is frequently limited, the observed association between cadaver dissection exposure and better academic performance suggests that maintaining access to cadaver-based practicals, even if limited, remains valuable [12]. The findings support continued efforts to improve cadaver procurement and preservation while exploring effective hybrid models that combine traditional dissection with virtual and model-based resources [13, 28]. Broader policy recommendations would require stronger evidence from multi-institutional and longitudinal studies.

The non-cadaveric group received a heterogeneous multimodal package. While these approaches improve accessibility and scalability, the significantly lower performance observed suggests that current multimodal substitution packages may not fully replicate the depth, tactile feedback, and three-dimensional relational understanding provided by active cadaver dissection [13, 19]. This supports arguments that cadaver-based methods should be preserved or prioritised where feasible, rather than replaced wholesale by alternatives that may not fully replicate the depth of learning required for safe medical practice [29]. This limitation is particularly relevant when considering complete replacement of cadaver-based teaching in low-resource settings.

One of the most valuable contributions of this study is its honest portrayal of the structural and practical realities confronting anatomy educators in cadaver-scarce settings. Cadaver scarcity forces departments to make difficult allocation decisions, often resulting in temporary educational inequity within the same student cohort. The constant struggle with inadequate infrastructure, funding, procurement challenges, and large class sizes is a daily reality for many anatomy departments across Nigeria and sub-Saharan Africa [26, 30, 31]. These challenges are rarely quantified in high-income country literature, making this context-specific evidence particularly novel and relevant to the global discourse on anatomy pedagogy.

### Limitations

This study has several limitations that should be considered when interpreting the results. As a comparative cross-sectional study based on naturally occurring groups due to cadaver scarcity, causality cannot be established. Although the groups were comparable in measured baseline characteristics (age, sex, and previous CGPA), unmeasured confounders such as study habits, motivation, and individual learning approaches may have influenced the findings. Group allocation was administrative rather than randomised, introducing potential selection bias. Additionally, access to supplementary resources (textbooks, atlases, and limited virtual tools) was not restricted, raising the possibility of cross-contamination between groups. Students in the non-cadaver group may also have had indirect cadaver exposure through peer interactions.

The study was conducted at a single institution and focused exclusively on extremity anatomy, limiting generalizability to other body regions and settings. The outcome measure relied on routine institutional examinations that, while blueprint-mapped to learning objectives, lacked formal psychometric validation (e.g., difficulty and discrimination indices) for this cohort. Furthermore, the study did not assess student satisfaction, perceptions, confidence, motivation, or comparative preferences between teaching modalities. Second-semester performance data following the crossover were not analysed. Finally, the heterogeneous nature of the non-cadaveric teaching package makes it difficult to isolate the effect of any single alternative modality.

## Conclusion

This comparative cross-sectional study found that first-year medical students with exposure to human cadaver dissection achieved higher gross anatomy examination scores than those taught using only non-cadaveric methods during the same semester. The cadaver dissection group demonstrated consistently better performance across MCQ, essay, and total scores.

These findings suggest that cadaver-based practical teaching remains a valuable component of gross anatomy education in resource-constrained settings such as ours. However, due to the observational design, potential confounders, and single-institution context, more rigorous multi-institutional and longitudinal studies are needed to confirm these observations and establish causality.

## Supplementary Information

Supplementary Material 1.

## Data Availability

The datasets generated and/or analysed during the current study are not publicly available due to institutional data protection policies and participant confidentiality, but are available from the corresponding author on reasonable request.
