# Supplementary material for: Gross anatomy performance associated with human cadaver dissection compared to non-cadaveric methods in nigerian medical students: a comparative cross-sectional study
Source: BMC Med Educ. 2026 May 29;26:1222. doi: 10.1186/s12909-026-09566-0 (PMC13422321; doi:10.1186/s12909-026-09566-0)

**APPENDIX**

**Appendix 1: Gross Anatomy Final Examination Questions (Extremities)**

This appendix contains the MCQs and essay questions that were used in the official first-year gross anatomy final examination for both groups in this study. The examination was part of the standard institutional assessment and was identical for all participants.

**Note**: The full examination consisted of 50 multiple-choice questions and several essay questions. All items shown below were administered to both Group A and Group B.


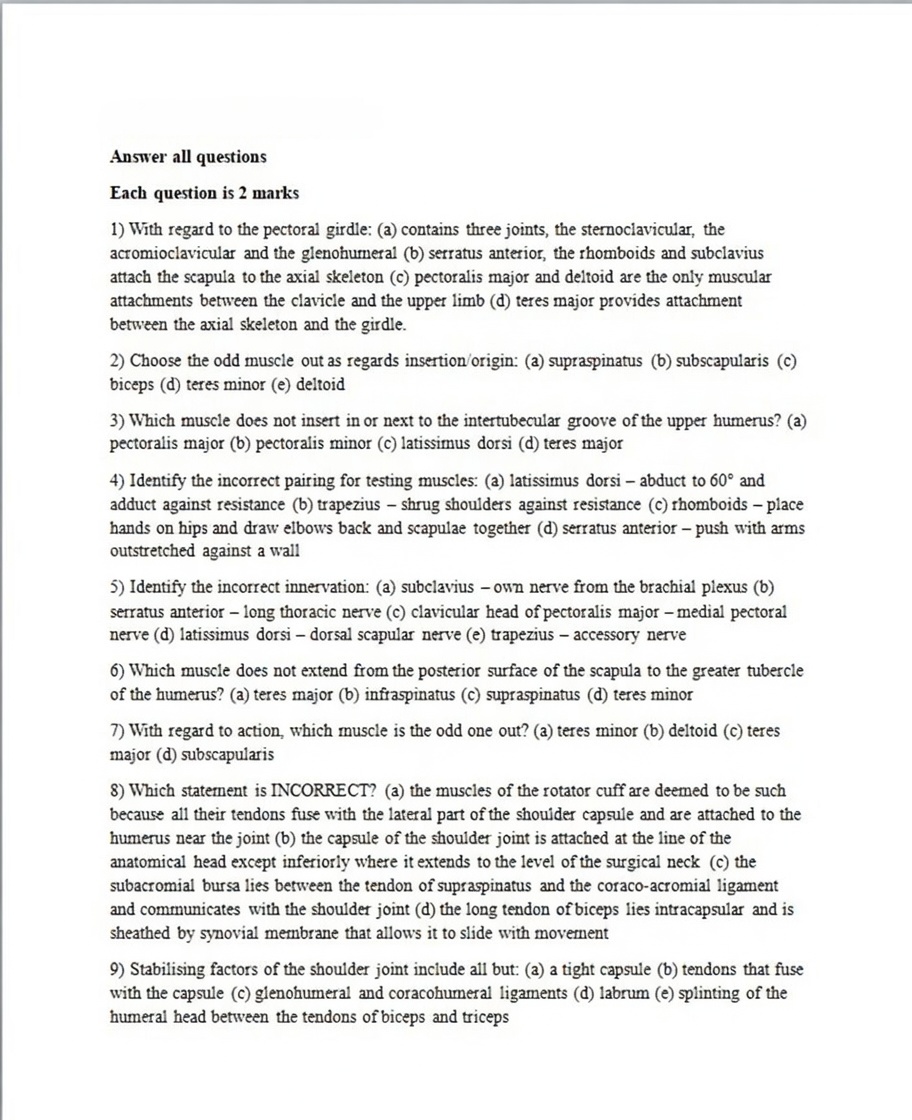


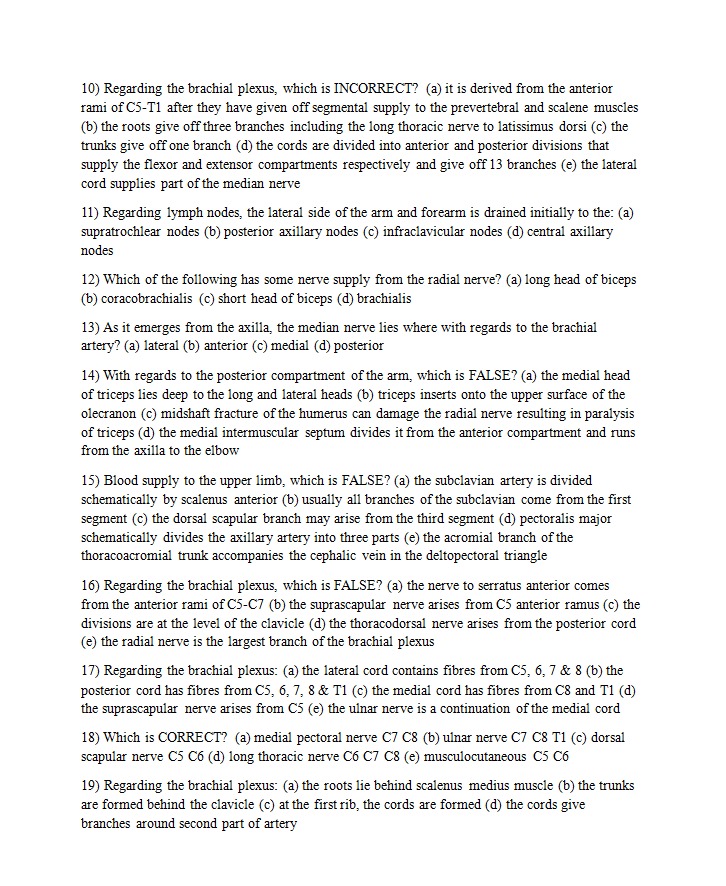


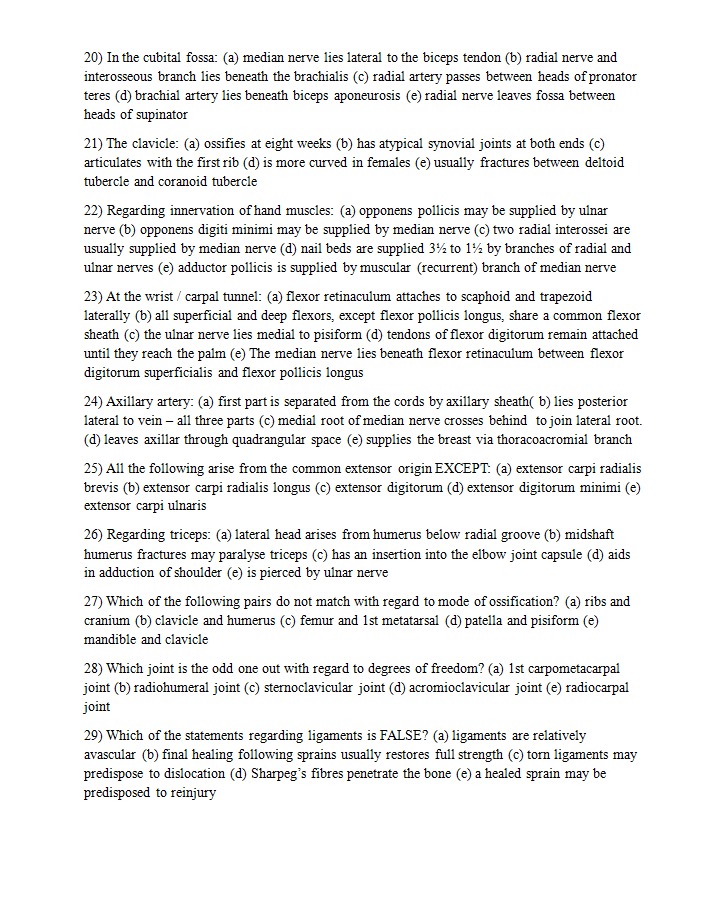


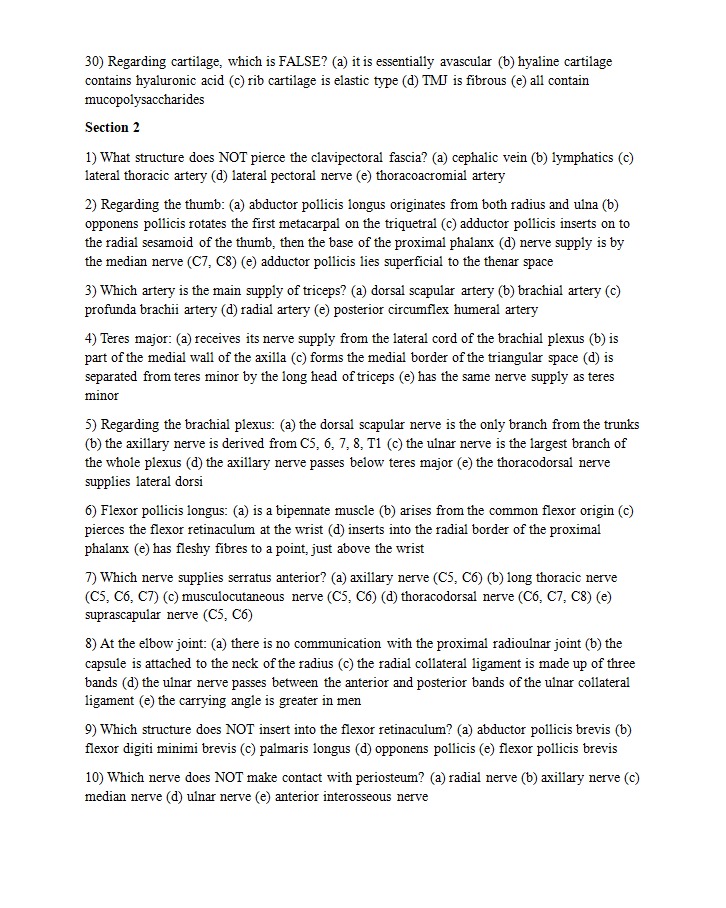


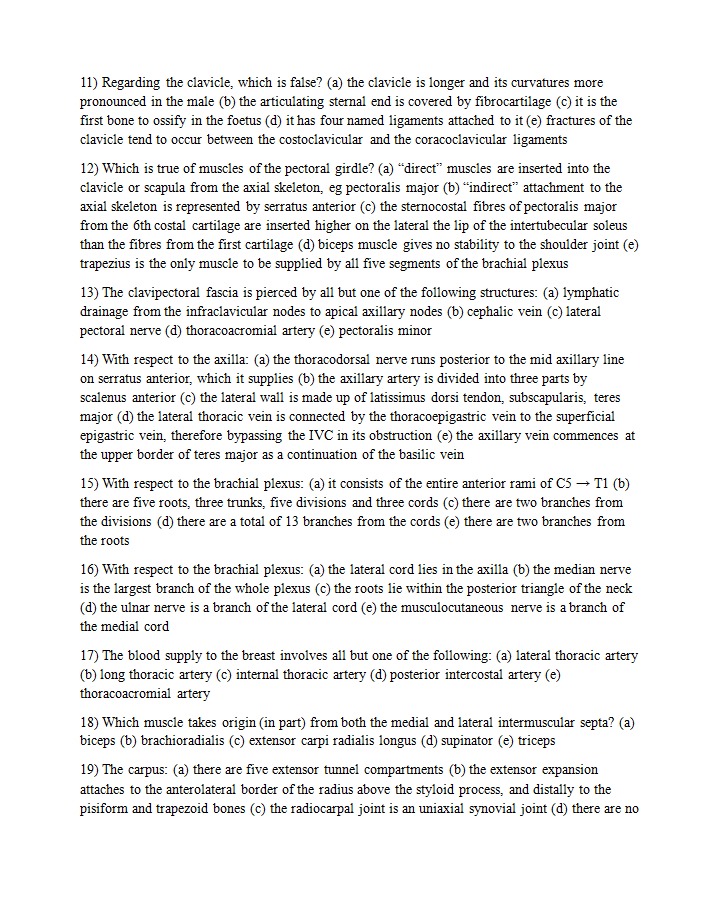


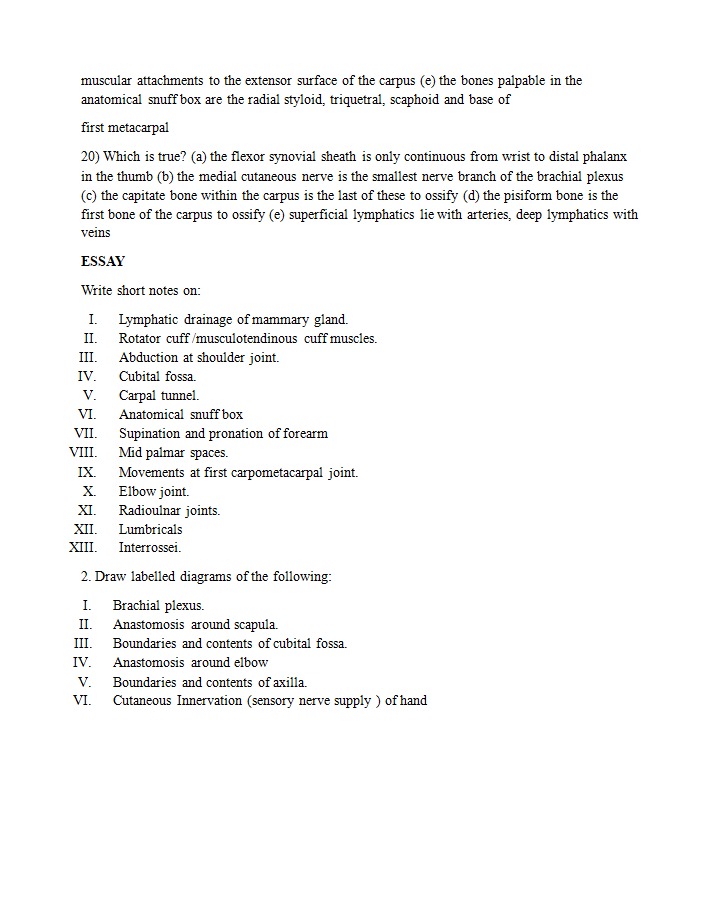

Supplement: Supplementary file 1 — Supplementary Material 1. [file 12909_2026_9566_MOESM1_ESM.docx]
